# Assessing the Cognitive Translational Potential of a Mouse Model of the 22q11.2 Microdeletion Syndrome

**DOI:** 10.1093/cercor/bhw229

**Published:** 2016-09-19

**Authors:** Simon RO. Nilsson, Kim Fejgin, Francois Gastambide, Miriam A. Vogt, Brianne A. Kent, Vibeke Nielsen, Jacob Nielsen, Peter Gass, Trevor W. Robbins, Lisa M. Saksida, Tine B. Stensbøl, Mark D. Tricklebank, Michael Didriksen, Timothy J. Bussey

**Affiliations:** 1 Department of Psychology, University of Cambridge, CambridgeCB2 3EB, UK; 2 Behavioural and Clinical Neuroscience Institute, University of Cambridge, CambridgeCB2 3EB, UK; 3 Department of Psychology, State University of New York at Binghamton, Binghamton, NY13902-6000, USA; 4 H. Lundbeck A/S, Synaptic Transmission, Neuroscience Research DK, Ottiliavej 9, Valby2500, Denmark; 5 In Vivo Pharmacology, Lilly Research Laboratories, Eli Lilly & Co. Ltd, Erl Wood Manor, Sunninghill Road, WindleshamGU20 6PH, UK; 6 Central Institute of Mental Health, Mannheim Faculty, University of Heidelberg, J5, 68159Mannheim, Germany

**Keywords:** animal model, cognition, copy number variation, 22q11.2 deletion syndrome

## Abstract

A chromosomal microdeletion at the 22q11.2 locus is associated with extensive cognitive impairments, schizophrenia and other psychopathology in humans. Previous reports indicate that mouse models of the 22q11.2 microdeletion syndrome (22q11.2DS) may model the genetic basis of cognitive deficits relevant for neuropsychiatric disorders such as schizophrenia. To assess the models usefulness for drug discovery, a novel mouse (Df(h22q11)/+) was assessed in an extensive battery of cognitive assays by partners within the NEWMEDS collaboration (Innovative Medicines Initiative Grant Agreement No. 115008). This battery included classic and touchscreen-based paradigms with recognized sensitivity and multiple attempts at reproducing previously published findings in 22q11.2DS mouse models. This work represents one of the most comprehensive reports of cognitive functioning in a transgenic animal model. In accordance with previous reports, there were non-significant trends or marginal impairment in some tasks. However, the Df(h22q11)/+ mouse did not show comprehensive deficits; no robust impairment was observed following more than 17 experiments and 14 behavioral paradigms. Thus – within the current protocols – the 22q11.2DS mouse model fails to mimic the cognitive alterations observed in human 22q11.2 deletion carriers. We suggest that the 22q11.2DS model may induce liability for cognitive dysfunction with additional “hits” being required for phenotypic expression.

## Introduction

Hemizygous microdeletion at chromosomal locus 22q11.2 is a copy number variation (CNV) that occurs in approximately 1:2000 births. It is associated with high incidence rates of cognitive impairment and neuropsychiatric disorders, including depression (Green et al. 2009), obsessive-compulsive disorder (Gothelf et al. 2004), attentional-deficit hyperactivity disorder and autism (Niklasson et al. 2002). The deletion is the strongest known genetic risk factor for schizophrenia with approximately 40% of carriers developing psychosis (Schneider et al. 2014).

Several genes within the deleted segment are involved in cortical development (Maynard et al. 2003) and some 22q11.2 deletion syndrome (22q11.2DS) carriers exhibit structural aberrations within regions involved in higher-order cognitive functioning such as the prefrontal cortex (PFC), hippocampus, and dorsal striatum (Campbell et al. 2006) as well as disrupted dopamine (DA) (Boot et al. 2007) and glutamate (da Silva Alves et al. 2011) signaling. Some of these structural abnormalities have also been reported in transgenic mouse models lacking the orthologous chromosomal segment (Ellegood et al. 2014).

22q11.2DS is associated with extensive cognitive impairments. 22q11.2DS is associated with mild to moderate reductions in IQ-scores and learning impairments are observed in 85–100% of carriers (Lipson et al. 1991). Carriers can show deficits in cognitive domains such as visuospatial memory (Bearden et al. 2001) working memory (Lajiness-O'Neill et al. 2005), attention (Bish et al. 2007) and cognitive flexibility (Lewandowski et al. 2006). Domain specific cognitive impairments also appear to be independent of general intelligence (Shapiro et al. 2014).

Several transgenic mouse models of 22q11.2DS have been created (Lindsay 2001) and cognitively phenotyped to varying extents by different research groups. Most encompass the full core region of the 22q11.1 deletion (Dgcr2-Hira), while others carry partial deletions (Table 1). Schizophrenia-relevant sensory and psychomotor behaviors have been reported as disrupted in several of these models. The Df(16)A^+/−^ mouse model has been shown to display baseline hyperactivity and an anxiogenic phenotype (Stark et al. 2008) while increased hyperactivity in response to MK-801 was reported in the Df/+ model (Kimoto et al. 2012). Similar to human carriers (Sobin et al. 2005) the Df(16)A^+/−^, Df1/+ and LgDel models displayed reduced prepulse inhibition (Paylor et al. 2001; Long et al. 2006; Stark et al. 2008) while the Df1/+ mutant had a increased acoustic startle response (Paylor et al. 2001).

**Table 1:** Cognitive functioning in the Df(h22q11)/+ mutant and other 22q11.2DS mouse models. ↓ impaired, ↑ improved,  ╳ no effect, − no data.

Model		Df(h22q11)/+	Df(16)A+/−	LgDel	Df1/+	
Deletion		Dgcr2-Hira	Dgcr2-Hira	Dgcr2-Hira	Dgcr14-Ufd1l	Znf74-Ctp
Strain		C57/Bl6NTac	C57/Bl6J	C57/Bl6N	Mixed C57/Bl6c-/c- ;129S5/SvEvBrd	129SvEvTac or mixed 129SvEvTac ; Crl:NIHBL(S)
Behavior	Paradigm					
Memory	Water maze <20 weeks	╳	−	−	╳^a^	−
	Water maze >20 weeks	╳	╳^b^	−	↓^a^	−
	Contextual fear conditioning	╳	↓^c^	╳^d^	↓^e^	╳^f^
	TUNL – pattern separation	╳	−	−	−	−
	Auditory-cue fear conditioning	╳	↓^c^	╳^d^	╳^e^	╳^f^
	Touchscreen PAL	╳	−	−	−	−
	Novel object recognition	╳	╳^g^	−	−	−
	Touchscreen discrimination learning					
	“Easy” discrimination	↑	−	−	−	−
	“Difficult” discrimination	╳	−	↓^h^	−	−
Working Memory	Y-maze spontaneous alternation	╳	−	−	−	−
	TUNL – delay challenge	↑	−	−	−	−
	Radial arm-maze	╳	−	−	−	−
	T-maze non-match to sample					
	Acquisition	↓	↓^c^ ╳^i^	−	↓^j^	−
	Delay challenge	╳	−	−	−	−
Executive function	PVT – Premature responses	╳	−	−	−	−
	5CSRTT – Premature responses	╳	−	−	−	−
	Touchscreen extinction learning	╳	−	−	−	−
	Touchscreen reversal learning					
	“Easy” reversal	↑	−	−	−	−
	“Difficult” reversal	╳	−	↓^h^	−	−
Attention	PVT – Reaction time	╳	−	−	−	−
	PVT – Correct responses	╳	−	−	−	−
	5-CSRTT – Accuracy	╳	−	−	−	−
	5-CSRTT – Omissions	↑	−	−	−	−

^a^Earls et al. 2010.

^b^Drew et al. 2011.

^c^Stark et al. 2008.

^d^Long et al. 2006.

^e^Paylor et al. 2001.

^f^Kimber et al. 1999.

^g^Fenelon et al. 2013.

^h^Meechan et al. 2015.

^i^Sigurdsson et al. 2010.

^j^Hughes et al. 2014.

In earlier studies, learning and memory deficits have been reported in 22q11.2DS mouse models. The Df(16)A^+/−^ and Df(16)1+ mutants (Stark et al. 2008; Hughes et al. 2014) had impaired spatial alternation learning in the T-maze although the impairment has not always been replicated (Sigurdsson et al. 2010). In the water maze, Df(16)1/+ mice (Earls et al. 2010) but not the Df(16)A^+/−^ mutant (Drew et al. 2011) showed age-dependent impairments. Recently, the LgDel mutant was shown to have improved early-phase reversal learning but impaired late-phase and overall touchscreen reversal learning as well as impaired discrimination performance (Meechan et al. 2015). The Df(16)A^+/−^ and the Df1/+ models (Paylor et al. 2001; Stark et al. 2008), but not the LgDel mouse (Long et al. 2006), also showed impaired fear conditioning. Normal contextual and auditory fear-conditioning was nevertheless observed in a 22q11.2DS model carrying a smaller 150 kB deletion (Kimber et al. 1999).

Didriksen et al. describes the development and basic characterization of a novel model of 22q11.2DS (Df(h22q11)/+) with a hemizygous deletion at the region on mouse chromosome 16 that correspond to the core region of the human 22q11.2 deletion (Didriksen et al. 2016). As expected, cortical mRNA expression of genes in the region is reduced approximately 50%. The model shows deficits in PPI and activity assays in response to stimulants in the absence of overt baseline motoric deficits, making it suitable for screening in assays of cognition.

Following on such findings, the current set of experiments investigated the extent and robustness of behavioral and cognitive deficits in this model with the ultimate aim of evaluating its potential for cognitive translational studies. To this end, four partners within the NEWMEDS collaboration (Innovative Medicines Initiative Grant Agreement No. 115008) have phenotyped the Df(h22q11)/+ mouse using an extensive battery of assays that depend on neural structures compromised in 22q.11.2DS and 22q.11.2DS mouse models (Ellegood et al. 2014). The test battery contained several touchscreen assays that parallel the computerized tasks used with patients (Bussey et al. 2012; Hvoslef-Eide et al. 2015) and are sensitive to deficits of rodent models of neuropsychopathology (Bartko et al. 2010; Brigman et al. 2010; Talpos et al. 2010; Graybeal et al. 2011; Romberg et al. 2011; Nithianantharajah et al. 2012, 2015; Gastambide et al. 2013; McAllister et al. 2013; Romberg et al. 2013; Kim et al. 2015). This represents the most extensive report of cognitive functioning in a 22q11.2DS transgenic mouse model to date (see Table 1).

## Method

### Animals

The generation of the Df(h22q11)/+ mouse is described in Didriksen et al. (2016). Df(h22q11)/+ mice were of c57BL/6NTac background. As 22q11.2DS models generated by others (Long et al. 2006; Stark et al. 2008), the hemizygous deletion of the Df(h22q11)/+ model extend between Dgcr2-Hira on mouse chromosome 16 and encompass orthologs of all functional genes on the critical human 22q11.2 locus with exception of clathrin heavy polypeptide-like 1 (*CLTCL*) which appears absent in the mouse genome (Botta et al. 1997). The mouse region also includes *Igll1* that is not part of the human region and has not previously been linked to behavior or cognition in the mouse. Transcriptional changes in the Df(h22q11)/+ model was confirmed by cortical RNAseq analysis and microarray analysis of gene products from the deleted segment; detected products were decreased to around 50% of the expression in WT mice (Didriksen et al. 2016).

**Figure 1.**
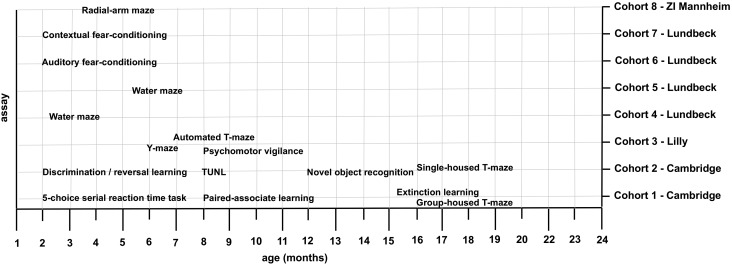
Age at the start of each experiment of 8 cohorts of Df(h22q11)/+ and WT littermates. Cohort 1 was tested on the 5-CSRTT, PAL, Extinction (all WT *N* = 16, TG *N* = 16) and T-maze (WT *N* = 10, TG N = 10). Cohort 2 was tested on reversal learning, TUNL, object recognition (all WT *N* = 16, TG *N* = 16) and T-maze (WT *N* = 11, TG N = 14). Cohort 3 was tested in the Y-maze (WT *N* = 15, TG *N* = 16), automated T-maze (WT *N* = 13, TG *N* = 16) and the rPVT (WT *N* = 13, TG *N* = 16). Cohort 4 (WT *N* = 16, TG *N* = 16) and Cohort 5 (WT *N* = 10, TG *N* = 8) were tested in the water maze. Cohort 6 (WT *N* = 24, TG *N* = 24) was tested in auditory fear conditioning. Cohort 7 (WT *N *= 20, TG *N* = 20) was tested on context-dependent fear conditioning. Cohort 8 (WT *N* = 16, TG *N* = 16) was tested in the radial-arm maze.

The experiments used 8 cohorts of animals. The age of animals at the start of each experiment and sample sizes for each experiment are shown in Figure 1 and the legend of Figure 1, respectively. Cohorts 1–5 and 8 consisted of male mice; cohorts 6–7 consisted of male and female mice. Animals were group-housed (with the exception of Cohort 2 and 8 which were single-housed prior to T-maze and radial arm maze experiments) with ad libitum access to water under stable temperature and humidity conditions. Deficits in acquisition of T-maze delayed non-match to sample has previously been reported in a single-housed 22q11.2DS mouse model. We therefore single-housed Cohort 2 for 7 weeks prior to the T-maze experiment to evaluate the role of housing condition in producing a phenotype in this task. Prior to behavioral training, animals were food deprived to about 85% of their free-feeding weight. Cohorts 1–2 and 8 were housed under a reversed 12 h light/dark cycle (lights on at 7 p.m.). Cohorts 3–7 were housed under a standard 12 h light/dark cycle (lights off at 7 p.m.). All studies were carried out in accordance with European Union regulation (directive 2010/63 of 22 September 2010), UK Animals (Scientific Procedures) Act 1986 and UK Animals (Scientific Procedures) Act 1986 Amendment Regulations 2012, and were approved by the Danish National Committee for Ethics in Animal Experimentation and German Committee on Animal Care and Use.

### Procedures

#### Attention and Behavioral Inhibition

##### Touchscreen 5-choice serial reaction time task (5-CSRTT)

All touchscreen assays were performed using 32 chambers (Campden Instruments, Loughborough, UK) with associated software (Abet II, Lafayette Instruments, Lafayette, IN, USA) described in detail elsewhere (Mar et al. 2013). The 5-CSRTT measures attention and impulsivity, cognitive domains disrupted in schizophrenia (Kahn et al. 2012) and 22q11.2DS (Lewandowski et al. 2006). The task is sensitive to attentional and impulsive disruptions in mouse models of neuropsychiatric disorders (Young et al. 2004; Hoyle et al. 2006; Romberg et al. 2011; Nilsson et al. 2016) and has been described extensively elsewhere (Romberg et al. 2011; Mar et al. 2013). Briefly, animals were trained to respond to a white-square stimulus using a 2 s stimulus durations (SD), 40 trial session length and 5 s delay. Once acquired, the animals were assessed on a series of probe tests. This included tests of decreased SDs (1.6, 1, 0.8, 0.6, 0.4, 0.2 s) increasing delays (7, 9, 11, 13 s) and tests of increased session length (140 trials) with decreasing SDs (1.6, 1, 0.8, 0.6 s). The main dependent variables where percent accuracy, percent omissions, percent premature responses, percent perseverative responses, correct response latency, and reward retrieval latency.

##### Rodent psychomotor vigilance task (rPVT)

The rPVT assesses impulsive control and is described elsewhere (Gastambide et al. 2013). Task performance is sensitive to disruptions in rodent models of schizophrenia (Gastambide et al. 2013). Briefly, a trial started with the illumination of the houselight (preparatory cue) followed by a variable interval (VI5s; range: 4–6 s) and the subsequent illumination of the magazine light for 10 s (imperative cue). Head entries into the magazine during the imperative cue were rewarded while head entries during the preparatory cue were scored as premature and resulted in a 5 s time-out. To assess impulsivity, probe-tests were performed at which length of the interval between the imperative and preparatory cues were extended from VI5s (range: 4–6 s) to VI10s (9–11 s) and VI15 s (14–16 s). The main dependent variable was percent premature responses. The number of correct responses, omissions and head entries, correct response latency and premature response latency was also collected for each mouse.

#### Cognitive Flexibility

##### Touchscreen discrimination and reversal learning

**Figure 2.**
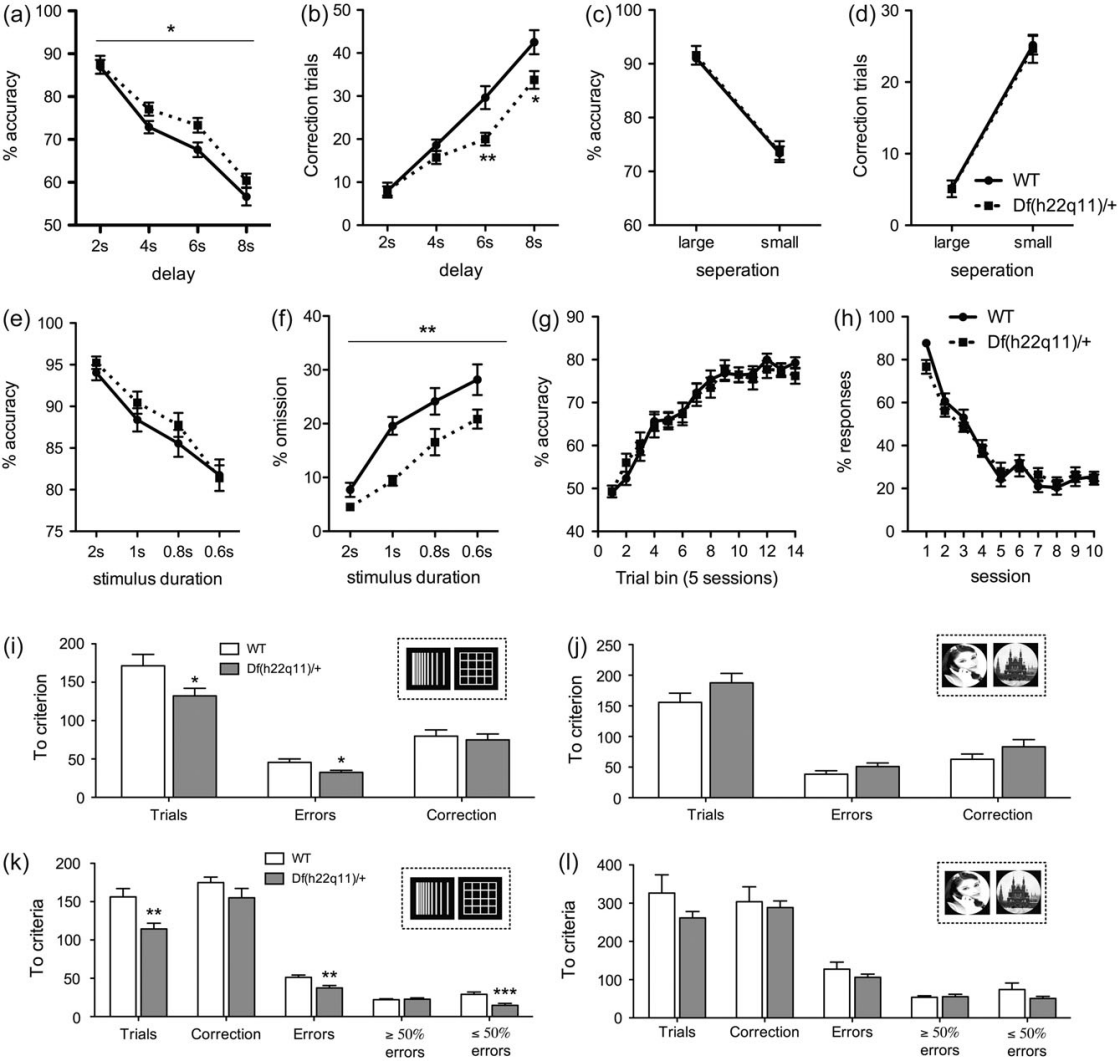
Performance of Df(h22q11)/+ and WT littermates on touchscreen assays. Data is presented as means ± SEM (*a*–*b*) TUNL – delay challenge. The Df(h22q11)/+ showed higher accuracies (*a*) and required fewer correction trials (*b*) at longer delays. This effect was reproducible (see Supplementary Fig. S4d–e). (*c*–*d*) TUNL – separation challenge. No effects of genotype. (*e*–*f*) 5-CSRTT. No effect on accuracy (*e*). Following extensive training (>100) sessions the Df(h22q11)/+ showed a duration-independent decrease in the number of omissions (*f*) when tested on 140-trial sessions. (*g*) PAL. No effects of genotype. (*h*) Extinction learning. No effect of genotype. (*i*–*l*) Discrimination learning and reversal learning. In the initial easy discrimination (i) and reversal challenge (k), the Df(h22q11)/+ showed improved learning. In a second more challenging discrimination (j) and reversal challenge (l) there were no effects of genotype. Insets depict the stimuli. Asterisk denote differences at which *p* < 0 .05 (*p < 0.05, **p < 0.01, ***p < 0.001).

Reversal learning measures cognitive flexibility which is disrupted in schizophrenia (Leeson et al. 2009) and 22q11.2DS (Lewandowski et al. 2006) and the LgDel mouse model (Meechan et al. 2015). The task is extensively described elsewhere (Horner et al. 2013; Mar et al. 2013). The task has been shown to be sensitive to transgenic mouse models schizophrenia (Nithianantharajah et al. 2012) and mouse PFC lesioning (Graybeal et al. 2011). Animals were tested on two separate discrimination/reversals, the first using more discriminable stimuli and the second using more challenging stimuli (see Fig 2*i–l*, insets). The criterion for successful discrimination and reversal learning was ≥80% accuracy for two consecutive sessions. The dependent variables were trials to criterion, errors to criterion, correction trials to criterion, average correct response latency, and average reward retrieval latency. During reversal learning, perseverative errors (the number of incorrect responses made before achieving >50% correct responding in session), learning errors (the number of incorrect responses made after achieving ≤50% correct responses in a session), and “perseveration index” (the number of correction trials as a ratio of errors) (Brigman et al. 2010) were calculated.

##### Touchscreen extinction learning

Extinction of appetitive responding is dependent on the PFC, striatum and amygdala (Quirk and Mueller 2007), areas disrupted in mouse models of 22q11.2DS (Ellegood et al. 2014). The task is extensively described elsewhere (Mar et al. 2013). In brief, animals were initially trained to respond to a single-white square stimulus within 10 s for reward. Animals were subsequently tested on extinction learning. During extinction learning, touches to the white-square stimulus resulted in the removal of the stimulus in the absence of rewards and conditioned reinforcers. The dependent variables were percent stimulus responses, response latency, and blank touches (during the ITI when no stimulus was shown).

#### Working and Short-Term Memory

##### Touchscreen delayed non-match to location (rTUNL)

The rTUNL task measures working memory and pattern separation, which are dependent upon the mPFC (McAllister et al. 2013) and hippocampus (Talpos et al. 2010). The rat version of the task is described elsewhere (Oomen et al. 2013). Here we use a simplified version of the task (Kim et al. 2015) (see Supplementary Methods). Animals were tested on separate tests of increasing delays (2, 4, 6, 8 s) and increasing stimuli separations (small, large). To assess possible genotype differences in the use of mediating strategies, animals were again tested on the 6 s delay with sessions being video-recorded and behaviors during the delay were scored and cost/benefit scores were analyzed (Talpos et al. 2010) using JWatcher (version 1.0). See supplementary methods for detailed protocol.

##### T-maze delayed non-match to location

One cohort was assessed in an automated version of the task (using 10, 30, 60 s delays). Two further cohorts were assessed in a hand-run version of the task using a protocol kindly shared by Joshua Gordon (Columbia University) (Stark et al. 2008; Sigurdsson et al. 2010). In the hand-run experiment, the first cohort remained group-housed while the second cohort was single-housed 7 weeks to the beginning of training (Võikar et al. 2004). Other groups have reported T-maze non-match to sample impairment in 22q11.2DS models using single-housed animals. The second cohort was therefore single-housed to evaluate the role of housing condition in a possible phenotype. See Supplementary Methods for detailed protocol.

##### Y-maze spontaneous alternation

The task is sensitive to rodent models of schizophrenia (Belforte et al. 2009) and is extensively described elsewhere (Dillon et al. 2008). The sequence or arms entered was recoded and analyzed by Ethovision XT 8.5 (Noldus, Nottingham, UK) during a 15 min session. The alternation rate was calculated as the number of alternations (e.g., ABC is an alternation, ABA is not) divided by the total number of alternation opportunities (i.e., the total number of arm entries minus two). The total distance traveled was also calculated for each mouse.

##### Radial-arm maze

The procedure is sensitive to mouse models of schizophrenia and is extensively described elsewhere (Inta et al. 2014). The main dependent variable was percent working memory errors, calculated as the number of re-entries into baited arms as a ratio of the number of arm entries.

#### Long-Term Memory

##### Touchscreen paired-associate learning (PAL)

The rPAL task measures visuospatial learning which is impaired in 22q11.2DS (Bearden et al. 2001) and schizophrenia (Barnett et al. 2010). The task is extensively described elsewhere (Horner et al. 2013). In rats, performance is impaired by hippocampal (Talpos et al. 2009) and mPFC (McAllister et al. 2013) lesions. Robust learning impairments in this task has been observed in an alternative transgenic mouse model of schizophrenia (Nithianantharajah et al. 2012). Animals were tested for 70 sessions and and the data was collapsed in 5-session bins for analyses. The dependent variables percent accuracy, correction trials, correct and incorrect response latencies.

##### Fear-conditioning

Impaired contextual and auditory fear-conditioning have been observed in a 22q11.2DS mouse model (Stark et al. 2008). The task is extensively described elsewhere (Medina et al. 2002).

##### Water maze

The procedure is extensive described elsewhere (Podhorna and Didriksen 2005). Separate cohorts of 10-week and 25-week old animals were tested with 4 and 6 acquisition days, respectively, followed by a probe trial 24 h later. Data were analyzed using Ethovision 3.0 (Noldus, Wageningen, The Netherlands).

##### Novel object recognition

The task has been described previously (Winters et al. 2008). Animals were tested on 3, 8, 14, and 24 h delays. Video analyses of behavior were made by an experimenter blind to the genotype and location of the novel object using JWatcher (version 1.0). For each animal, the discrimination ratio (time spent exploring the novel object divided by the total time spent exploring), object exploration time in the sample-phase, and object biases were calculated.

### Statistical Analyses

Data was analyzed through one-way ANOVA with genotype as between-subjects variable or repeated-measures ANOVA with genotype (and sex, where appropriate) as between-subject variable(s) and session, delay, stimulus duration, learning phase or stimulus separation as the within-subjects variable. Significant interactions where followed by one-way ANOVA. See Result for experiment-specific information. Analyses were done using SPSS (v21.0, IBM Corp, Armonk, NY).

## Results

See Table 1 for a summary of the results in the complete test battery. See Table S1 for overall average response latencies and reward retrieval latencies during touchscreen cognitive tests.

### Attention and Behavioral Inhibition

#### 5-CSRTT

The data was analyzed by mixed model ANOVAs. In short sessions with decreasing SDs (Fig. S1a–b) there was no effect of genotype on accuracy (genotype: *F*_1,30_ = 0.240, *p* = 0.877; genotype × SD: *F*_6,180_ = 1.141, *p* = 0.340) or omissions (genotype: *F*_1,30_ = 0.235, *p* = 0.631; genotype × SD: F_6,180_ = 0.591, *p* = 0.737). There were no effects of genotype or genotype × SD interaction on any other behavioral measure (all *p* ≥ 0.193). When increasing the delay, there was no effect of genotype on premature responses (Fig. S1c; genotype: *F*_1,30_ = 0.240, *p* = 0.878; genotype × SD; *F*_4,120_ = 1.102, *p* = 0.359). There were no other effects of genotype or genotype × SD interactions (all *p* ≥ 0.055). Tests on long sessions (140 trials) following extensive training (>100 session) showed an improved performance in the Df(h22q11)/+ with the transgenic making fewer omissions (Fig. 2*e–f*; genotype: *F*_1,30_ = 12.134, p = 0.002). On response latencies, there was a genotype × SD interaction (*F*_3,90_ = 6.467, *p* < 0.001) with Df(h22q11)/+ animals showing faster responses at the 2 s SD (*F*_1,30_ = 5.384, *p* = 0.027; data not shown). There were no other effects of genotype or genotype × SD interactions (all *p* ≥ 0.287).

#### Rodent Psychomotor Vigilance Task

**Figure 3.**
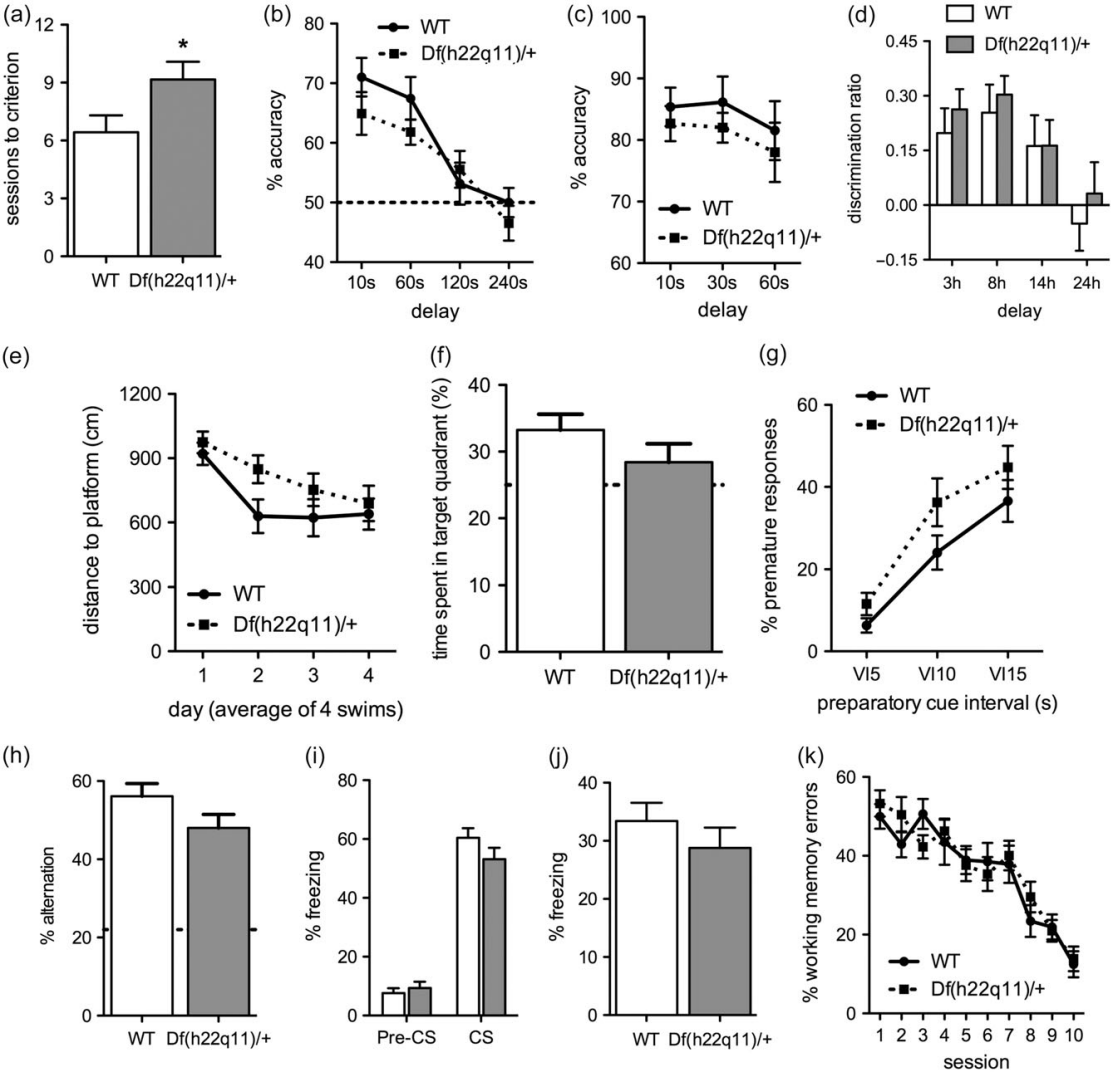
Performance of Df(h22q11)/+ and WT littermates on non-touchscreen assays. Data is presented as means ± SEM. (*a*–*b*) Hand-run T-maze. Df(h22q11)/+ mice required more sessions to acquire T-maze alternation criterion (*a*). No effect of genotype on tests of variable delays (*b*). Broken line represents random responding. (*c*) Automated T-maze. No effects of genotype. (*d*) Novel object recognition. No effects of genotype. (*e–f*) Water maze. No significant genotype differences in 10-week old animals on task acquisition (*e*) or a 24 h probe test (*f*). No effects were observed in 10-week old animals (see Supplementary Fig. S4a-b). (*g*) Psychomotor vigilance task. No effect of genotype on probe-tests of impulsive-like behavior. (*h*) Y-maze. No effects of genotype. Broken line represents random responding. (*i*) Auditory fear conditioning. No effect of genotype (*j*) Context-dependent fear conditioning. No effect of genotype. (*k*) Radial arm-maze. No effect of genotype. Asterisk denote differences at which *p* < 0.05.

There was no effect of genotype on premature responses during task acquisition (Fig. S2a–c; Mixed model ANOVA, effect of genotype: *F*_1,27_ = 0.483, *p* = 0.493; interaction effect genotype × session *F*_6,162_ = 0.703, *p* = 0.648). There were no effects of genotype on accuracy, omissions or average correct response latencies (Mixed model ANOVA or between-subject ANOVA, all *p* ≥ 0.333). Increasing the variable interval revealed a non-significant trend for the Df(h22q11)/+ mutant to make more premature responses than the WT (Fig. 3*g*; Mixed model ANOVA, effect of genotype: *F*_2,54_ = 3.805, *p* = 0.062; interaction effect genotype × interval: *F*_2,54_ = 0.375, *p* = 0.689).

### Learning and Cognitive Flexibility

#### Visual Discrimination and Reversal Learning

The data was analyzed by one-way between-subjects ANOVAs. In the first discrimination, the Df(h22q11)/+ mice required fewer trials (Fig. 2*i*; *F*_1,30_ = 4.793, *p* = 0.036) and made fewer incorrect responses (*F*_1,30_ = 6.101, *p* = 0.019). Genotype did not affect correction trials or latency measures (all *p* ≥ 0.345). In the first reversal (Fig. 2*k*), the mutant required fewer trials (*F*_1,30_ = 11.769, *p* = 0.002) and incorrect responses to criterion (*F*_1,30_ = 9.771, *p* = 0.004). Df(h22q11)/+ mice made fewer late errors (*F*_1,30_ = 12.556, *p* = 0.001) but not early errors (*F*_1,30_ = 0.98, *p* = 0.757). Again, genotype failed to affect correction trials and latency measures (all *p* ≥ 0.172). A second test using less discriminable stimuli showed no effect of genotype on discrimination (Fig. 2*j*) or reversal learning (Fig. 2*l*; all *p* ≥ 0.127).

#### Extinction Learning

Genotype did not affect extinction learning (Fig. 2*h*; Mixed model ANOVA, effect of genotype: *F*_9,270_ = 0.118, *p* = 0.734; interaction effect genotype × session: *F*_9,270_ = 1.820, *p* = 0.065).

### Working and Short-Term Memory

#### Water Maze

In 10-week old animals (Fig. 3*e–f*), there was no effect of genotype on acquisition (Mixed model ANOVA, effect of genotype: *F*_1,30_ = 2.763, *p* = 0.107; interaction effect genotype × session: *F*_3,90_ = 0.856, *p* = 0.467) or delay-probe (Between-subjects ANOVA, *F*_1,30_ = 1.754, *p* = 0.195). There was also no effect of genotype in 25-week old animals (Fig. S4a–b. all *p* ≥ 0.180).

#### Y-maze Spontaneous Alternation

There was no effect of genotype on alternation rate (Fig. 3*h*; Between-subjects ANOVA, *F*_1,29_ = 2.869, *p* = 0.101).

#### Radial-arm Maze

There was no effect of genotype on working memory errors over 10 test session (Fig. 3*k*; Mixed model ANOVA, effect of genotype: *F*_1,30_ = 0.282, *p* = 0.599; interaction effect genotype × session: *F*_9,270_ = 0.740, *p* = 0.672).

#### Trial-unique Non-match to Sample

The Df(h22q11)/+ showed higher accuracies relative to the WT when manipulating the delay (Fig. 2*a*; Mixed model ANOVA, effect of genotype: *F*_1,30_ = 5.567, *p* = 0.025; interaction effect genotype × delay: *F*_3,90_ = 1.090, *p* = 0.357). Df(h22q11)/+ mice required fewer correction trials (Fig. 2*b*; Mixed model ANOVA, interaction effect genotype × delay: *F*_3,90_ = 4.094, *p* = 0.009) at the 6 s and 8 s delays (Between-subjects ANOVAs, *p* ≤ 0.017). Tests of pattern separation showed no effect of genotype (Fig. 2*c–d*; all *p* ≥ 0.832). In video analyses of mediating behaviors, Df(h22q11)/+ mice again outperformed WTs at the 6 s delay (Fig. S4d–e; Mixed model ANOVA, effect of genotype on accuracy: *F*_1,30_ = 4.879, *p* = 0.035; correction trials: *F*_1,30_ = 7.879, *p* = 0.009). There were no genotype differences in cost/benefit scores for any behavior (Fig. S3).

#### T-maze Delayed Non-match to Location

There was no effect of housing-condition on performance (all *p* ≥ 0.509) and groups were collapsed for analyses. In acquisition, the Df(h22q11)/+ mouse showed slower learning relative to the WT (Fig. 3*a*; Between-subjects ANOVA, effect of genotype: *F*_1,43_ = 4.579, *p* = 0.038). Genotype did not affect performance in tests of 10 s, 60 s, 120 s, and 240 s delays (Fig. 3*b*; Mixed model ANOVA, effect of genotype: *F*_1,43_ = 1.719, *p* = 0.197; interaction effect genotype × delay: *F*_3,129_ = 0.864, *p* = 0.462). Further tests using 10 s and 90 s delays revealed no genotype differences (Fig. S4c). There were no effects of genotype in the automated T-maze task (Fig. 3*c*; all *p* ≥ 0.372).

#### Fear-conditioning

The data was analyzed by mixed model ANOVAs. In the auditory cue condition, there were no effects of genotype on freezing (Fig. 3*i*; genotype, pre-CS: *F*_1,46_ = 0.405, *p* = 0.528; CS: *F*_1,46_ = 2.046, *p* = 0.159). Males were more immobile than females (data not shown; sex, Pre-CS: *F*_1,46_ = 5.835, *p* = 0.020; CS: *F*_1,46_ = 4.501, *p* = 0.039). Separate analyses by sex showed no effect of genotype (*p* ≥ 0.280). In the context condition, there were no effects of genotype (Fig. 3*j*; *F*_1,38_ = 0.975, *p* = 0.330) or sex (*F*_1,38_ = 1.904, *p* = 0.176).

### Long-Term Memory

#### Paired Associate Learning

Data was analyzed by mixed model ANOVAs. There was no effect of genotype on accuracy (Fig. 2*g*; genotype: *F*_1,26_ = 0.679, *p* = 0.417; genotype × session: *F*_13,338_ = 0.481 *p* = 0.935), correction trials (genotype: *F*_1,26_ = 0.236, *p* = 0.631; genotype × session: *F*_13,390_ = 0.528 *p* = 0.907; data not shown) or latency measures (*p* ≥ 0.406).

#### Novel Object Recognition

Genotype had no effect on novel object recognition at any delay (Fig. 3*d*; Mixed model ANOVA, effect of genotype: *F*_1,24_ = 0.244, *p* = 0.625; interaction effect genotype × delay: *F*_3,72_ = 0.057, *p* = 0.982).

## Discussion

This report represents a multi-site evaluation of cognitive functioning in a 22q11.2DS mouse model by several partners in the NEWMEDS collaboration. It is the most comprehensive evaluation of cognition in a 22q11.2DS mouse model to date (Table 1). We selected a set of behavioral tests that have been shown to be sensitive to manipulations relevant for the 22q11.2DS and schizophrenia. These assays each evaluates individually distinct yet on a broader level partially overlapping cognitive functions and have demonstrated sensitivity to manipulations of PFC, hippocampal, DA and glutamate systems, as well as transgenic mouse models of neuropsychiatric disorders, including schizophrenia (Brigman et al. 2008; Winters et al. 2008; Talpos et al. 2009, 2010; Graybeal et al. 2011; Nithianantharajah et al. 2012; Gastambide et al. 2013; McAllister et al. 2013).

We found that the Df(h22q11)/+ mutant, in agreement with previous reports on 22q11.2DS models, shows impairments or trends towards impairments in several behavioral assays relevant to the cognitive impairments of neuropsychiatric disorders. This includes a deficit in acquisition of a T-maze non-match-to-sample task as well as non-significant trends towards impaired spatial memory in the Y-maze and water maze. The Df(h22q11)/+ mouse also showed trends towards increased impulsive responding in the psychomotor vigilance task and impaired auditory cue fear-conditioning.

However, the Df(h22q11)/+ mice showed normal performance in a range of other assays. This includes normal performance on object recognition, paired-associates learning, extinction learning, and contextual fear-conditioning. Improved performance was observed on cognitive flexibility (reversal learning), attention (5-CSRTT), and touchscreen spatial working memory (TUNL). In sum, although the Df(h22q11)/+ mouse model has been shown to recapitulate certain sensory and psychomotor phenotypes with relevance for schizophrenia (Didriksen et al. 2016), and minor cognitive deficits were observed in this study, it fails to mimic the extent of cognitive impairment observed in human 22q11.2 deletion carriers and schizophrenia patients. Considering possible reasons for the apparent mismatch between the CNS changes and the broad lack of behavioral effects, it is most unlikely that it results from a lack of statistical power or sensitivity of the behavioral assays, as these assays have revealed deficits using other lines of genetically modified mice (Brigman et al. 2008, 2010; Bartko et al. 2011; Romberg et al. 2011; Barkus et al. 2012; Coba et al. 2012; Nithianantharajah et al. 2012; Ryan et al. 2012; Romberg et al. 2013; Nithianantharajah et al. 2015). It remains possible that functional compensatory changes protect the animals from critical neuronal molecular effects and profound cognitive impairments. Such compensation is not obviously present in 22q11.2DS and schizophrenia patients. On the other hand, it is also possible that the neurobiological changes seen in the model are insufficient to produce profound deficits. It is important to keep in mind that the 22q11.2 microdeletion has incomplete penetrance in humans and syndrome expression presumably depends on the complete genetic makeup in concert with environmental influences. Thus, the Df(h22q11)/+ mouse may more appropriately be thought of as a liability model rather than a disease model.

Earlier studies indicate that 22q11.2DS models with overlapping heterozygote deletions showed impaired (Stark et al. 2008; Hughes et al. 2014) or trends towards impaired (Sigurdsson et al. 2010) spatial learning in the T-maze. In agreement with these earlier studies, we saw impaired acquisition learning in this task. This mild effect was transient, however; when mice were subsequently tested on the same delay used during acquisition, there was no impairment. Furthermore, the impairment could not be re-instated by challenging the animals further with longer delays. The ability of Df(h22q11)/+ mice to perform well on longer delays suggests that these animals do not have a working memory impairment per se; instead the initial mild deficit, which was present on day one of testing, may be more related to the propensity of animals to non-match, or other nonspecific performance factors. There were no significant effects of genotype in the automated version of the task. The data therefore do not convincingly support that the Df(h22q11)/+ mouse combined with T-maze testing might serve as a useful model for assessing the effects of cognitive enhancing drugs on working memory.

We observed trends toward impairments in auditory fear-conditioning and in the water maze when tested at 10 and 25 weeks of age. Previous studies of 22q11.2DS models have reported inconsistent results in these tasks. The Df(16)A^+/−^ and Df1/+ mice (Paylor et al. 2001; Stark et al. 2008) had deficits in contextual fear-conditioning following a 24 h delay. The Df(16)A^+/−^ mouse (Stark et al. 2008) also showed reduced auditory fear-conditioning. However, the Df1/+ mouse had normal auditory fear-conditioning and normal contextual fear-conditioning using a 1 h delay (Paylor et al. 2001). Similarly, no auditory or contextual fear conditioning deficits were present in the LgDel mouse (Long et al. 2006) or in mice with the smaller *Znf74-Ctp* deletion (Kimber et al. 1999). In the water-maze, 16–20 weeks old Df(16)1/+ mice but not 6–8 week old Df(16)1/+ mice showed spatial learning deficits (Earls et al. 2010) and no impairment was observed in Df(16)A^+/−^ mutants at 5–7 months of age (Drew et al. 2011).

The Df(h22q11)/+ mutant showed improved discrimination and reversal learning when challenged with an easily discriminable stimulus pair, yet no effect of genotype was observed when tested using a less discriminable stimulus pair. In contrast, the LgDel mouse was shown to display impaired touchscreen discrimination and reversal learning (Meechan et al. 2015). Meechan et al. (2015) observed large performance variability in their LgDel mutants relative to WT animals. Here, many transgenic animals displayed behavior comparable to WTs and the overall impairment on reversal learning appeared driven by a subgroup of mutants. There are as yet no reports of such heterogeneity in the Df(16)1/+ , Df(16)A^+/−^ or Df1/+ mutants, and we did not observe it in Df(h22q11)/+ mice. Furthermore, although the LgDel mouse showed an increase in overall incorrect responses, the mutant also showed a decrease in early perseverative-like errors, most relevant for modeling prefrontal dysfunction and cognitive inflexibility impairment of psychiatric disorders, including schizophrenia (Iversen and Michkin 1970; Elliott et al. 1995; Dias et al. 1996). Other factors that may explain the contrasting behavioral phenotypes of the LgDel and Df(h22q11)/+ models (or other behavioral discrepancies observed across laboratories in the independently generated 22q11.2DS mouse models) include the different background strains, ages, sex, and possible protocol differences, including the use of different stimuli, housing conditions and training protocols.

The Df(h22q11)/+ mouse also showed improved performance in the TUNL test of working memory and the 5-CSRTT test of attention (following extensive training). A tentative suggestion is that these effects may be produced by COMT haploinsuffiency. Cognitive abilities are related to a PFC-DA inverted-U curve whereby low levels of increased stimulation at the D_1_ receptor can improve performance (Kroener et al. 2009). In both humans and rodents, COMT inhibition can improve cognitive flexibility, attention, and working memory (Tunbridge 2004; Lapish et al. 2008). Decreased COMT activity through the COMT val^108/158^ allele has been associated with elevated cortical DA (Lachman et al. 1996), improved cognitive flexibility (Malhotra et al. 2002), working memory and attention (Blasi 2005; Caldú et al. 2007). COMT heterozygous mice have shown increased PFC-DA levels (Gogos et al. 1998), improved attentional set-shifting (Scheggia et al. 2014) and working memory (Papaleo et al. 2008). Elevated PFC-DOPAC is also observed in the Df(h22q11)/+ mouse (Didriksen et al. 2016). Mouse and human COMT orthologs also show radical differences in enzymatic activity (Chen et al. 2004) which may be related the divergent cognitive phenotypes of 22q11.2 deletion carriers and the Df(h22q11)/+ mouse. Thus, improved performance on tests of executive functioning in the Df(h22q11)/+ mutant as well as the lack of effect of genotype in many assays could be related to COMT heterozygosity and increased PFC-DA levels overshadowing putative detrimental effects of further allelic insufficiencies on cognition in this model.

### The 22q11DS Mouse Model as a Translational Tool for Psychiatric Drug Discovery

Being the strongest known genetic risk factor for schizophrenia, the 22q11.2DS is of considerable interest for translational research as it may allow for a model with good construct validity when probing the genetic basis of schizophrenia-relevant cognitive impairments. Our findings nevertheless suggest that deletion of the relevant chromosomal segment in the mouse confer a mild phenotype that fails to mimic the extent of the human cognitive impairment. There are differences within the orthologous regions in mice (MMU16) and humans (22q11.2) that may be involved in the lack of translation of the complete cognitive phenotype. This includes several genomic rearrangements in 22q11.2 segment relative to the mouse MMU16, and the low-copy repeats of the 22q11.2 region thought to infer segment instability is absent on the rodent MMU16 (Botta et al. 1997). The mouse do not carry a CLTCL ortholog for which hemizygosity has been linked to some primarily non-cognitive 22q11.2DS phenotypes (Sirotkin et al. 1996; Holmes et al. 1997). Mouse and human COMT orthologues also differ in enzymatic activity (Chen et al. 2004). Furthermore, in other constitutive mouse models of schizophrenia, haploinsufficiency can be insufficient in producing cognitive phenotypes of the disorders and homozygous mutants have been required (Nithianantharajah and Grant 2013). Homozygous 22q11.2DS mouse models are, however, unfortunately not presently viable. As stated above, the 22q11.2DS model may also be considered a liability model. Thus a more pronounced phenotype might appear in other assay conditions or in combination with additional environmental risk factors. For example, pubertal stress revealed latent neuropathological consequences of prenatal immune activation, another risk factor for schizophrenia (Giovanoli et al. 2013). It may be that a similar approach is necessary for unmasking robust dysfunctions in the 22q11.2DS mouse model and other CNV models (Kogan et al. 2015; Nilsson et al. 2016) where phenotypic expression within multiple domains of cognition can remain below threshold.

Yet it should be noted that current study is not a complete assessment of 22q11.2DS-relevant cognitive functions. For example, 22q11.2DS is associated with deficits in visual sustained attention as measured by Go/no-go or continuous performance tasks (Lewandowski et al. 2006; Antshel et al. 2010; Shashi et al. 2010, 2012; Hooper et al. 2013; Schoch et al. 2014). Several 22q11.2DS mouse models (Kimber et al. 1999; Paylor et al. 2001; Long et al. 2006; Stark et al. 2008), including the Df(h22q11)/+ mutant (Didriksen et al. 2016), also show sensorimotor gating deficits, considered a marker of pre-attentative information processing (Powell et al. 2009). Although the current battery did not include tests of visual sustained attention or motivational functioning, the demonstrated link between these domains and 22q11.2DS may warrant further behavioral study in the Df(h22q11)/+ mouse.

## Conclusion

In agreement with the previous literature, the mouse model of 22q11.2DS showed some impairments or trends towards impairments in tasks of cognition. However, this extensive test battery indicates that the model does not mimic the extent of 22q11.2DS-related cognitive deficits as seen in affected human carriers. It may be that functional compensations protect the animals from profound cognitive deficits or that the genetic changes observed in this mutant are insufficient to produce major behavioral impairments unless they are exposed to additional risk factors. Possibilities remain that tests not included in the current study are sensitive to the genetic disruptions of the 22q.11.2DS model.

## Supplementary Material

Supplementary material can be found at: http://www.cercor.oxfordjournals.org/

Supplementary Data
