# Supplementary material for: Assessing the Cognitive Translational Potential of a Mouse Model of the 22q11.2 Microdeletion Syndrome
Source: Cereb Cortex. 2016 Sep 19;26(10):3991–4003. doi: 10.1093/cercor/bhw229 (PMC5028007; doi:10.1093/cercor/bhw229)
Supplement: Supplementary Data [file supp_26_10_3991__index.html]

Assessing the Cognitive Translational Potential of a Mouse Model of the 22q11.2 Microdeletion Syndrome — Assessing the Cognitive Translational Potential of a Mouse Model of the 22q11.2 Microdeletion Syndrome — Supplementary Data 

# Assessing the Cognitive Translational Potential of a Mouse Model of the 22q11.2 Microdeletion Syndrome

## Supplementary Data

Supplementary Data

- Supplementary Data - docx file
