# Supplementary material for: Assessing the Cognitive Translational Potential of a Mouse Model of the 22q11.2 Microdeletion Syndrome
Source: Cereb Cortex. 2016 Sep 19;26(10):3991–4003. doi: 10.1093/cercor/bhw229 (PMC5028007; doi:10.1093/cercor/bhw229)
Supplement: Supplementary Data [file supp_bhw229_22q11_cognition_supplementary_revised_2.docx]

**SUPPLEMENTARY MATERIAL**

**Assessing the cognitive translational potential of a mouse model of the 22q11.2 microdeletion syndrome**

Simon RO. Nilsson^1,2^(PhD), Kim Fejgin^3^(PhD), Francois Gastambide^4^(PhD), Miriam A. Vogt^5^(PhD), Brianne A. Kent^1,2^(PhD), Vibeke Nielsen^3^(BSc)_,_ Jacob Nielsen^3^(PhD), Peter Gass^5^(MD), Trevor W. Robbins^1,2^(PhD), Lisa M, Saksida^1,2^(PhD), Tine B. Stensbøl^3^(PhD), Mark D. Tricklebank^4^(PhD), Michael Didriksen^3^(PhD)_­­_, Timothy J. Bussey^1,2^(PhD).

^1^Department of Psychology, University of Cambridge, Cambridge, CB2 3EB, UK. ^2^Behavioural and Clinical Neuroscience Institute, University of Cambridge, Cambridge, CB2 3EB, UK. ^3^H. Lundbeck A/S, Synaptic Transmission, Neuroscience Research DK, Ottiliavej 9, Valby 2500, Denmark. ^4^In Vivo Pharmacology, Lilly Research Laboratories, Eli Lilly & Co. Ltd, Erl Wood Manor, Sunninghill Road, Windlesham, GU20 6PH, UK. ^5^Central Institute of Mental Health, Mannheim Faculty, University of Heidelberg, J5, 68159, Mannheim, Germany.

*Corresponding author:* Simon Nilsson, Department of Psychology, State University of New York at Binghamton, Binghamton, NY 13902-6000, USA. Email: sn440@cam.ac.uk. Tel: +1 (203) 444 6821.

SUPPLEMENTARY METHOD

*Touchscreen delayed non-match to location (TUNL) protocol*

*Task acquisition*. The screen was arranged into a horizontal row of five response locations using a 5-hole mask. A session began with the onset of the magazine-light. A nosepoke in the magazine extinguished the magazine-light and caused the onset of a sample-phase where a white-square stimulus was presented in one out of two possible response windows in the five-hole mask (either the leftmost or the rightmost location). A nosepoke response to the white-square caused the stimulus to disappear, onset of the magazine-light, a 1s tone, onset of a 2 s delay and a 33% likelihood of reward delivery. A third of the sample trials were rewarded to encourage continuous responding and reduce the use of mediating strategies. After the delay, a test-phase was initiated where two white-square stimuli were presented on the screen (both the leftmost or the rightmost location). If the animal responded to the stimulus not presented during the sample-phase, a correct response was recorded, the stimuli were removed, the magazine-light turned on, and reward was delivered coupled with a 1s tone. A 15s ITI was initiated once the animal retrieved the reward. If the animal responded to the stimulus presented in the sample-phase, an incorrect trial was recorded, the stimuli were removed from the screen, and the houselight was turned on for a 5s time-out after which the 15s ITI was initiated. An incorrect response was always followed by a correction trial were the sample and test stimuli were presented in the same locations as the previous trial. Correction trials did not count towards the session trial count. All animals received 14 sessions of training under these parameters. The animals were subsequently assessed in separate tests of spatial working memory and pattern separation. This was followed by additional tests using longer delays to assess the use of mediating behaviours through video analyses. The dependent variables were percent accuracy, number of correction trials, number of completed trials, average correct response latency and reward retrieval latency.

*Spatial working memory.* Animals received three probe sessions of increasing delays (4s, 6s, 8s). Each test session lasted for two consecutive sessions and the data presented is the mean for those two sessions. Prior to each probe test, the animals received four consecutive sessions on the baseline 2s delay parameter.

*Pattern separation.* In the sample-phase, the white-square stimulus was presented in any one of the five different response-windows. In the test-phase, the stimuli were presented closer in proximity such as the target and sample stimuli always were presented with the separation of one ‘blank’ response window. The delay remained as 2s throughout the probe sessions. Each test session lasted for two consecutive sessions and the data presented is the mean for those two sessions.

*Behavioural analyses.* To assess genotype differences in the use of mediating strategies, animals were video-recorded when receiving an additional 6s delay probe and behaviours during the delay was scored using JWatcher (version 1.0). Analyses of cost or benefits of ten types of behaviours were analysed using a modified protocol of that presented used in rats (Talpos et al. 2010, Neurobiol Learn Mem. 94:341–352). Briefly, for each behaviour, we first calculated the number of trials the behaviour did not occur. This number of trials was then multiplied by the number of trials the animal displayed the behaviour to produce an ‘excepted’ number of correct trials should the behaviour not occur. This value was then deducted from the number of correct trials the animals made while performing the behaviour. These values were then expressed as a percentage of cost or benefit on accuracy should that behaviour occur on every performed trial.

The behaviours and definitions of behaviours analysed were: *orienting towards the back of the box* – body oriented towards the magazine with the head tilted to the to-be correct or to-be incorrect location. *Orienting towards the screen -* body oriented towards the centre of the screen with the head tilted to the to-be correct or to-be incorrect location. *Orienting towards chamber-side -* body oriented and/or rearing towards the side of the chamber with the head tilted to the to-be correct or to-be incorrect location. *Orienting body in the magazine –* the mouse enters the magazine in a tilted position with its body oriented to the to-be correct or to-be incorrect location. *Waiting in-front of response-location –* the mouse spends >1s or more of the delay sitting 2cm or less in-front of the to-be correct or to-be incorrect location.

Hand-run T-maze delayed non-match to location protocol

The mice were assessed using a protocol kindly shared by Joshua Gordon (Columbia University). Two separate cohorts of animals at 17 months of age were tested on the T-maze. The first cohort remained group-housed, while the second cohort was single-housed 7 weeks prior to the beginning of habitation (Võikar et al. 2004, Genes Brain Behav. 4:240–252).

*Habituation*. Animals initially received two days of maze habituation. On the first day of habituation, all three arms were baited with a single sucrose reward pellet (14mg, Sandown Scientific, Middlesex, UK) and the animal was placed in the start-arm with the start-box door closed. Once the animal had consumed the pellet in the start-box, the door was removed and the animal was free to explore the maze. Once the animal had consumed the two remaining pellets, the start-arm was re-baited. When the animal had consumed the pellet in the start-arm, the two choice-arms were again baited. On the second day of habituation, the start-box door was closed whilst the choice-arms were re-baited. The two sessions lasted for 10min each.

*Shaping*. Next, all animals received two days of shaping using one open choice-arm with the second choice-arm being blocked. The animal was placed in the baited start-arm with the door lowered. Once the animal had consumed the pellet, the start-door was removed. Once the animal consumed the pellet in the end of the open choice-arm, the start-arm was baited. When the animal returned to the start-arm, the door was closed and the choice-arm alternated with the previously blocked arm now being open and he previously open arm now being blocked. This continued for 10 trials (with one trial being a left and right forced choice) with the order of the open and closed arms presented in a pseudorandom order.

*Acquisition*. Acquisition training on delayed non-match to location began on the fourth day. Again the mouse was placed in the baited start-box. The animal initially received a forced-choice sample-trial with one pseudorandomly open arm. Once the animal consumed the pellet, the start-arm was baited. When the animal returned to the baited start-arm, the start-door was closed, the choice-point was wiped with a disinfectant and the choice-arm block was removed. After a delay (approximately 10s), the start-door was opened and the mouse could now enter both the left or right choice-arm. Once the animal entered a choice-arm, the opposite arm was immediately closed. When the animal reached the end of the choice-arm the start-arm was then re-baited and the next trial began once the animal had re-entered the start-box. The acquisition criterion was 7 correct responses over 10 trials for three consecutive days.

*Probe-trial.* Immediately after achieving acquisition criterion the animals were tested in a single probe-trial that pitted the use of an egocentric strategy against the use of exteroceptive allocentric cues. Here, the animals received a standard sample-trial after which the maze was rotated 180°. An animal entering same arm in the sample- and test-trials would indicate the use of an allocentric strategy while entering the opposite arm in the sample- and choice phases would suggest the use of an egocentric response strategy. Both arms were baited in the choice-trial in order not to disrupt the formed association between response and reinforcement.

*Variable delays.* Next, animals were tested using variable delays over 4 sessions of 12 trials. Each session consisted of 3 trials at 10s, 60s, 120s, and 240s delays. Across the four sessions, animals received 12 trials at each delay in a pseudorandom order identical for each mouse counterbalanced across sample-arm locations. Presented data represents the mean percent correct over the 12 trials at each delay. Animals in the 2^nd^ (single-housed group) also received an additional test of variable delays at 10s and 90s for three consecutive sessions at 12 trials per session.

**SUPPLEMENTARY FIGURES**

**Figure S1.** Initial tests in the 5-CSRTT. **(a) Accuracy.** No effect of genotype (F_1,30_ = 0.024, p = 0.877) or genotype × SD interaction (F_6, 180_ = 1.143, p = 0.340)**. (a) Omissions.** No effect of genotype (F_1,30_ = 0.235, p = .631) or genotype × SD interaction (F_6, 180_ = 0.591, p = 0.737)**. (a) Premature responses.** No effect of genotype (F_1,30_ = 0.024, p = 0.878) or genotype × delay interaction (F_4, 120_ = 1.102, p = 0.359)**.**

**Figure S2.** Acquisition performances on the psychomotor vigilance task. **(a) Premature responding.**  No effect of genotype (F_1,27_ = 0.483, p = 0.493) or genotype × session interaction (F_6, 162_ = 0.703, p = 0.648). **(b) Response latency.** No effect of genotype (F_1,27_ = 0.022, p = 0.882) or genotype × session interaction (F_6, 162_ = 0.378, p = 0.892). **(c) Omissions.** No effect of genotype (F_1,27_ = 0.407, p = 0.529) or genotype × session interaction (F_6, 162_ = 1.427, p = 0.191).

**Figure S3.** Analyses of mediating behaviours during the 6s delay in TUNL. Asterisk denote significant cost or benefit on accuracy score at which p < .05 (*p < .05, **p < .01, ***p <.001). No significant genotype differences within any behaviour (all p ≥ .063).

**Figure S4.** Performance **of** Df(h22q11)/+ mice and WT littermates in the water maze when 24 weeks old (a-b), in hand-run T-maze delayed non-match to location using variable 10s and 90s delays (c) and in the TUNL task during video analyses (d-e). **(a) Water maze acquisition.** Main effect of session (F_5, 80_ = 11.82, p < 0.0001) but no effect of genotype (F_1,16_ = 1.962, p = 0.180) or genotype × separation interaction (F_1,16_ = 0.475, p = 0.501). **(b) Water maze 24-delay probe.** No effect of genotype (F_1,16_ = 1.214, p = 0.287). **(c) T-maze, 10s vs 90s delays.** No effect of genotype (F_1, 22_ = 0.571, p = 0.458) or genotype × delay interaction (F_1, 22_ = 0.360, p = 0.555). **(d-e) TUNL performance during video analyses.** Main effect of genotype (F_1,30_ = 5.568, p = 0.025) but no genotype × delay interaction (F_1,30_ = 1.893, p = 0.179) on accuracy. Main effect of genotype (F_1,30_ = 8.607, p = 0.006) and genotype × delay interaction (F_1,30_ = 5.06, p = 0.032) on correction trials. Asterisk denote genotype differences at which p < 0.05.

|  | **Genotype** | |  |
| --- | --- | --- | --- |
| **Task** | **WT** | **Df(h22q11)/+** | ***p*** |
| **PAL** |  |  |  |
| Correct response latency | 1.59±0.04 | 1.65±0.06 | 0.406 |
| Incorrect response latency | 1.70±0.03 | 1.78±0.08 | 0.304 |
| Reward retrieval latency | 1.18±0.03 | 1.20±0.05 | 0.769 |
| **Visual discrimination 1 (‘easy’)** |  |  |  |
| Correct response latency | 3.09±0.15 | 3.28±0.21 | 0.468 |
| Reward retrieval latency | 1.58±0.15 | 1.77±0.14 | 0.345 |
| **Reversal learning 1 (‘easy’)** |  |  |  |
| Correct response latency | 3.92±0.36 | 3.99±0.43 | 0.909 |
| Reward retrieval latency | 1.45±0.06 | 1.50±0.09 | 0.667 |
| **Visual discrimination 2 (‘difficult’)** |  |  |  |
| Correct response latency | 1.96±0.09 | 2.02±0.10 | 0.663 |
| Reward retrieval latency | 1.26±0.04 | 1.21±0.07 | 0.538 |
| **Visual discrimination 2 (‘difficult’)** |  |  |  |
| Correct response latency | 2.54±0.12 | 2.41±0.11 | 0.395 |
| Reward retrieval latency | 1.39±0.09 | 1.26±0.06 | 0.232 |
| **TUNL – delay challenge** |  |  |  |
| Correct response latency | 1.90±0.14 | 2.23±0.22 | 0.110 |
| Incorrect response latency | 2.11±0.20 | 2.27±0.26 | 0.630 |
| Reward retrieval latency | 1.19±0.03 | 1.24±0.04 | 0.261 |
| **TUNL – separation challenge** |  |  |  |
| Correct response latency | 2.31±0.11 | 2.80±0.25 | 0.083 |
| Incorrect response latency | 3.02±0.28 | 3.24±0.39 | 0.652 |
| Reward retrieval latency | 1.24±0.13 | 1.19±0.04 | 0.678 |
| **Extinction – response acquisition** |  |  |  |
| Response latency | 1.67±0.13 | 1.89±0.17 | 0.303 |
| Reward retrieval latency | 1.03±0.04 | 1.02±0.04 | 0.721 |
| **Extinction learning** |  |  |  |
| Response latency | 4.23±0.13 | 4.32±0.09 | 0.570 |
| **5CSRTT – SD probes (2–0.25s, 140 trials)** |  |  |  |
| Correct response latency | 1.09±0.03 | 1.06±0.04 | 0.605 |
| Reward retrieval latency | 1.15±0.03 | 1.13±0.03 | 0.641 |
| **5CSRTT – delay probes (5-13s)** |  |  |  |
| Correct response latency | 1.14±0.03 | 1.10±0.03 | 0.350 |
| Reward retrieval latency | 1.24±0.03 | 1.24±0.04 | 0.996 |

**Table S1.** Average response latencies and reward retrieval latencies in **of** Df(h22q11)/+ mice and WT littermates during touchscreen cognitive assays.
